# The Role of Rituximab in ABO-Compatible Renal Transplantation: A Comprehensive Systematic Review and Meta-Analysis of Randomized Controlled Trials

**DOI:** 10.3390/medicina62040636

**Published:** 2026-03-27

**Authors:** Albaraa Y. Alotaibi, Razique Anwer, Shouq F. Alhazmi, Abdullah M. Almousa, Abdulrahman A. Alsughayyir, Ghala M. Alhmidani, Waad S. Alzahrani, Hassan A. Alsufayan, Mohamed H. Taiara, Rayan T. Alanazi, Hamad S. Alanazi, Rayan K. Alfandi, Hussam A. Mansaki

**Affiliations:** 1 College of Medicine, Imam Mohammad Ibn Saud Islamic University (IMSIU), Riyadh 13317, Saudi Arabia; 444001072@sm.imamu.edu.sa (A.Y.A.); abdualmousa20@gmail.com (A.M.A.); abdulrahmanalsughyyirr@gmail.com (A.A.A.); ghala.al.2020@gmail.com (G.M.A.); alsufayanhassan@gmail.com (H.A.A.); riuu1696@gmail.com (R.T.A.); hmad.saleh612@gmail.com (H.S.A.); rayanaf7726@gmail.com (R.K.A.); 2 Department of Pathology, College of Medicine, Imam Mohammad Ibn Saud Islamic University (IMSIU), Riyadh 13317, Saudi Arabia; 3 Pharmaceutical Care Department, King Faisal Specialist Hospital & Research Centre, Madinah 42523, Saudi Arabia; shoqfahd@gmail.com; 4 Department of Internal Medicine, King Faisal Medical Complex, Taif 26514, Saudi Arabia; waadaledwani@hotmail.com; 5 College of Medicine, Sulaiman Alrajhi University, Al Bukayriyah 52726, Saudi Arabia; hmztayar1998hmo@gmail.com; 6 College of Medicine, King Abdulaziz University, Jeddah 22254, Saudi Arabia; hosaamabdulah@gmail.com

**Keywords:** ABO-compatible renal transplantation, rituximab, graft survival, acute graft rejection

## Abstract

*Background and Objectives*: Rituximab, a monoclonal antibody targeting CD20+ B-cells, is used in autoimmune diseases and B-cell malignancies. Recently, rituximab has been used as an induction agent in kidney transplantation. Our study evaluates the efficacy of rituximab induction on biopsy-proven acute rejection (BPAR), patient survival, and graft survival in ABO-compatible patients. *Materials and Methods*: A systematic literature search was performed across five databases, following PRISMA guidelines, and the protocol was registered in PROSPERO (CRD42024603984). The inclusion criteria consisted of randomized controlled trials (RCTs), exploring the impact of rituximab induction on eligible kidney transplant recipients with BPAR, graft, and patient survival as the primary outcomes. Risk ratios (RR) with 95% confidence intervals (CI) were estimated by the random-effects model. The risk of bias was assessed via the Cochrane ROB-2 tool across five domains. *Results*: From an initial 859 potentially relevant studies, three RCTs, including 454 patients, met the inclusion criteria. At 6 months, rituximab was associated with a non-significant reduction in the risk of BPAR (RR 0.76; 95% CI, 0.50–1.15; I^2^ = 0%). Similarly, patient survival remained unchanged at 6 months (RR 1.01; 95% CI, 0.98–1.04; I^2^ = 0%). Due to significant heterogeneity, long-term outcomes were assessed using narrative synthesis, which revealed no additional benefit for rituximab in terms of graft or patient survival. Furthermore, potential safety concerns—especially in terms of infection risk—were noted. The certainty of this evidence was low, due to clinical heterogeneity in the included studies causing very serious indirectness. *Conclusions*: Induction with rituximab provides no significant benefit over standard regimens. Thus, its routine use in ABO-compatible renal transplants may not be justified. The available evidence is of low certainty. Further trials are needed before drawing a firm conclusion and to determine the best dosage, timing, and combinations.

## 1. Introduction

B cells—precursors of plasma cells—can discharge many cytokines to boost cellular immunity. They are also excellent antigen-presenting cells, making them significant factors in alloimmunity [1,2]. The growing need for renal transplantation has led to the discovery of innovative uses for certain medications, such as rituximab [3]. Rituximab is a chimeric monoclonal antibody directed toward CD20, a specific transmembrane protein expressed in all mature B cells except plasma cells [4,5]. Rituximab induces B lymphocyte depletion, allowing for complete elimination in 88% of transplant recipients, with an effect that lasts approximately 15 months [5,6]. Consequently, patients may experience side effects, including late-onset neutropenia that is secondary to the administration of rituximab [7,8]. The FDA approved the application of rituximab for several disorders, including grade I non-Hodgkin’s lymphoma and rheumatoid arthritis, and it is still being researched for other applications, such as in renal transplantation [9]. In transplantation settings, rituximab is administered preoperatively as a desensitizing agent, perioperatively to prevent the production of donor-specific antibodies, and postoperatively to avoid antibody-mediated rejection (ABMR) [10,11,12,13,14]. Even in the absence of splenectomy, studies have reported promising outcomes in renal transplantation [5,15]. This is especially true for ABO-compatible transplants, which show superior results across multiple metrics, including markedly lower infectious mortality compared to ABO-incompatible transplants (27% vs. 82%, respectively), as well as reduced rates of ABMR and post-surgical hemorrhage [16,17].

However, in the absence of a living donor, receiving an ABO-compatible transplant from a deceased donor can necessitate an extended duration of dialysis, potentially exceeding 10 years. Alongside that, waitlist mortality is high; according to the Kaplan–Meier method, mortality on the waiting list may approach 45%, while a competing risk model shows a lower rate of 12% [18]. Recent studies of rituximab’s effect have been contentious. In 2016, a study involving 38 patients revealed almost no evidence of a significant improvement in renal function between the rituximab and placebo groups [19]. Conversely, a study conducted in 2015 with 281 patients found that biopsy-proven acute rejection (BPAR) rates for rituximab and a placebo were 16.7% and 21.1%, respectively. Moreover, in patients with a high immunological risk, rituximab showed a risk reduction in BPAR to 17.9% compared to 38.2% in the placebo group. Furthermore, the rituximab group was more likely to experience less ABMR and a lower incidence of delayed graft function in the immunologically high-risk subgroup [20].

Although rituximab has been the topic of multiple studies, its effectiveness and safety in renal transplantation remain questionable, due to a lack of randomized control trials (RCTs). Therefore, further research is required to address this gap. In this systematic review and meta-analysis, we conducted a comprehensive search for all available relevant studies aimed at investigating the effect of adding rituximab on patient survival, graft survival, and acute graft rejection in ABO-compatible patients.

## 2. Methods

### 2.1. Ethics and Registration

In this review, we followed the PRISMA (Preferred Reporting Items of Systematic Reviews and Meta-Analysis) guidelines to increase the clarity, transparency, quality, and value of this review [21]. This review is registered with the International Prospective Register of Systematic Reviews (PROSPERO) with the following register ID: CRD42024603984 [22].

### 2.2. Literature Review

A comprehensive literature search was conducted across several databases, including PubMed, Google Scholar, Web of Science, ScienceDirect, Ovid Medline, ClinicalTrials.gov, ISCRTN and ICTRP. Additional articles were identified from the references of the reviewed articles. The search strategy incorporated key terms such as “Rituximab” OR “anti-CD20” OR “B-lymphocyte depleting agent”, AND “Induction therapy” OR “Immunosuppression” OR “Pre-transplantation treatment”, AND “Renal transplantation” OR “Kidney transplant” OR “Graft survival”. We evaluated the studies based on the PICO (population, intervention, comparison, outcome) criteria.

### 2.3. Methodology for Selecting Studies

All studies included in this review met the following criteria: (1) published in English; (2) included adult or pediatric patients undergoing ABO-compatible renal transplantation; (3) evaluated rituximab-based induction therapy compared with standard induction protocols without rituximab or alternative induction therapies; (4) reported at least one primary outcome of interest—patient survival, graft survival, or acute rejection rates, or reported a secondary outcome of interest—safety-related outcomes, including malignancies, leukopenia, infection, and cardiovascular events; (5) were designed as randomized controlled trials; (6) no time restrictions were applied. Studies were excluded based on the following criteria: (1) studies including ABO-incompatible renal transplantation, (2) studies without clearly defined rituximab dosing or regimen for induction, (3) studies comparing different B-cell depleting agents without a rituximab arm, (4) studies that did not report data of interest, (5) studies not published in English, and (6) non-randomized studies, case reports, small case series, and non-peer-reviewed abstracts or letters.

### 2.4. Process of Screening and Data Extraction

#### 2.4.1. Screening

Articles were screened concurrently and independently by three reviewers, with a ‘blind on’ feature, using the Rayyan collaboration platform (Rayyan Systems Inc., Cambridge, MA, USA; available at: https://www.rayyan.ai/) [23]. Disagreements were resolved by a fourth reviewer. Following that, the full texts of the included articles were reviewed collaboratively by three reviewers. Ultimately, three unique trials were included as follows: (1) the study by Sautenet et al., which includes a long-term follow-up published separately by Bailly et al.; (2) the study by van den Hoogen et al.; and (3) the study published by Tydén et al. in 2009, which comprises a short-term study complemented by a long-term follow-up published in a separate paper [6,12,19,20,24].

#### 2.4.2. Data Extraction

An extraction form was used by two reviewers to record the following variables: (1) the total number of patients, (2) the number of patients in the intervention/rituximab group, (3) the number of patients in the control/placebo group, (4) the number of patients who did not undergo the treatment, (5) the outcomes being measured (e.g., complications, quality of life, etc.), (6) the mean age of patients in intervention/rituximab group (in years) along with the standard deviation, (7) the mean age of patients in the control/placebo group (in years) along with the standard deviation, (8) the number and percentage of male patients in the total cohort, (9) the number and percentage of male patients in the intervention/rituximab group, (10) the number and percentage of male patients in the control/placebo group, (11) the number and percentage of female patients in the total cohort, (12) the number and percentage of female patients in the intervention/rituximab group, (13) the number and percentage of female patients in the control/placebo group, (14) the BMI (kg/m^2^) of patients in the control/placebo group and in the intervention/rituximab group—mean and standard deviation, (15) etiologies of end-stage renal disease (ESRD) (16) the number of patients who are undergoing their first kidney transplant, (17) the number of patients who had one or more previous kidney transplants, (18) the number of deceased donors, (19) the number of living donors, (20) the total number and percentage of patients with donor-specific antibodies, (21) the number of patients in the rituximab group with antibodies targeting Class I HLA antigens (and their percentage), (22) the number of patients in the control group with antibodies targeting Class I HLA antigens (and their percentage), (23) the number of patients in the rituximab group with antibodies targeting Class II HLA antigens (and their percentage), (24) the number of patients in the control group with antibodies targeting Class II HLA antigens (and their percentage), (25) antigen mismatch data for the rituximab group, (26) antigen mismatch data for the control group, (27) a panel-reactive antibody for the rituximab group, and (28) a panel-reactive antibody for the control group. Intention-to-treat (ITT) data were prioritized for extraction; however, per-protocol (PP) data were extracted if the trials exclusively reported their outcomes based on this analysis. In cases of data discrepancies between an original publication and its extended follow-up report, the quantitative meta-analysis was restricted to the verified short-term data from the original trial, while the long-term data were assessed via narrative synthesis to prevent introducing bias.

### 2.5. Assessment of Quality and Bias Risk

The risk of bias was assessed by two reviewers, using the Cochrane risk of bias (ROB2) tool to evaluate the quality of RCTs [25]. This tool includes five domains, each targeting a specific source of bias, categorized as follows: bias arising from the randomization process, bias due to deviations from the intended interventions, bias due to missing outcome data, bias in the measurement of the outcome, and bias in the selection of the reported result. Signaling questions aim to elicit information that is relevant to an assessment of risk of bias, which can be judged using an algorithm based on the following: low (low risk of bias for all domains), some concerns (for at least one of the domains), or high (at least one domain has a high risk or some concerns for multiple domains). Publication bias was not reliably assessed, due to the small number of included trials (*n* = 3). Funnel plot asymmetry is not considered a reliable indicator of publication bias when fewer than 10 studies are available. We acknowledge this limitation, as it is consistent with Cochrane’s guidance, which recommends caution when interpreting funnel plots [26].

### 2.6. Certainty of Evidence Assessment

The certainty of evidence for each reported outcome was assessed using the Grading of Recommendation, Assessment, Development, and Evaluation (GRADE) framework provided by the Cochrane Collaboration [27]. All the included studies were initially rated as ’high’ certainty, as all of them were RCTs. We assessed each outcome for possible certainty downgrading, based on five domains: (1) risk of bias, (2) inconsistency, (3) indirectness, (4) imprecision, and (5) publication bias. The final certainty result was rated as high, moderate, low, or very low.

### 2.7. Statistical Analysis

Review Manager software (RevMan) version 5.4 (The Cochrane Collaboration, London, UK) was used for statistical analysis. Event numbers were used for dichotomous variables to calculate the risk ratios (RRs) and a 95% confidence interval (CI). In addition, to determine the heterogeneity of the studies, I^2^ and X^2^ were performed. The I^2^ statistic was interpreted according to guidelines suggested by the Cochrane Collaboration: 0–40% (might not be important), 30–60% (may represent moderate heterogeneity), 50–90% (may represent substantial heterogeneity), and 75–100% (considerable heterogeneity). The outcomes included for the analysis were as follows: 1. patient survival, 2. graft survival, 3. biopsy-proven rejection, 4. bacterial infection, 5. CMV infection, and 6. leukopenia incidence. To prevent chronological bias, quantitative pooling was restricted to short-term time points (6 months). Long-term outcomes with highly variable follow-up durations were evaluated using a narrative synthesis approach. A random-effect model using the DerSimonian and Laird (DL) estimator was adopted, as it is the standard method in RevMan 5.4. Statistical significance was defined as a two-sided *p*-value of less than 0.05. Given the limited number of trials in this review (k = 3), we acknowledge that the DL method could underestimate the between-study variance; therefore, these pooled results should be interpreted with caution.

## 3. Results

### 3.1. The Literature Findings

**Figure 1:**
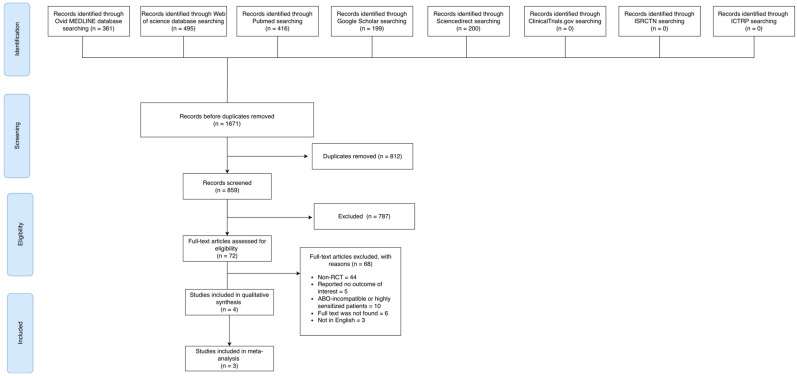
Flowchart (PRISMA) of the literature search and study selection process.

A search of eight databases, namely Ovid MEDLINE, Web of Science, PubMed, Google Scholar, ScienceDirect, ClinicalTrials.gov, ISRCTN, and ICTRP, yielded a total of 859 potentially relevant articles. After screening by abstract and title, 787 articles were excluded, as they did not meet the criteria. The remaining 72 were retrieved and assessed for eligibility. Among them, 68 were excluded because they were not RCTs (*n* = 44), did not report an outcome of interest (*n* = 5), included ABO-incompatible or highly sensitized patients (*n* = 10) or for other reasons (*n* = 9). A total of three RCTs comprising four published reports published between 2009 and 2020, with a total of 454 patients, met our inclusion criteria and were included in our review (Figure 1). The RITUX-ERAH trial was represented by two separate publications detailing 1-year and 7-year follow-up data [6,19]. Table 1 summarizes the main characteristics of the included studies, and Table 2 compares the characteristics of the included patients across studies.

### 3.2. Patient-Reported Outcomes, Complications, and Clinical Outcomes

#### 3.2.1. Short-Term Graft and Patient Survival

**Figure 2:**
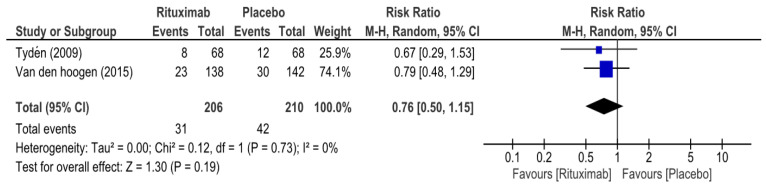
Forest plot of biopsy-proven acute rejection (at 6 months) [12,20].

**Figure 3:**
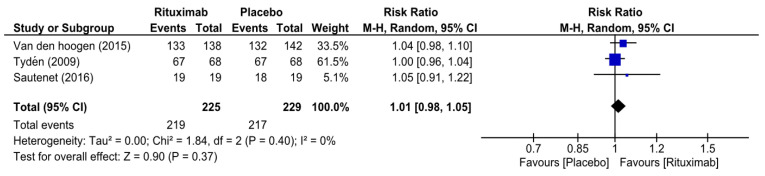
Forest plot of graft survival (at 6 months) [12,19,20].

**Figure 4:**
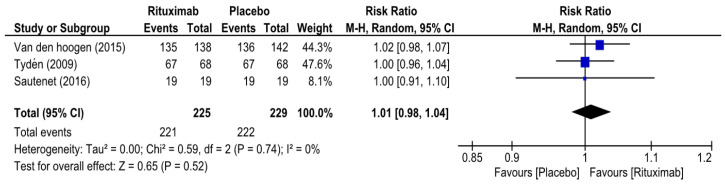
Forest plot of patient survival (at 6 months) [12,19,20].

At 6 months, the overall risk ratio (RR) of biopsy-proven acute rejection (BPAR) in patients who received rituximab was 0.76 (95% CI 0.50–1.15, I^2^ = 0%) (Figure 2), while the overall RR of graft survival in the rituximab group at 6 months was 1.01 (95% CI 0.98–1.05, I^2^ = 0%) (Figure 3). The overall RR of patient survival at 6 months in the rituximab group was 1.01 (95% CI 0.98–1.04, I^2^ = 0%) (Figure 4). Because the RITUX-ERAH trial by Sautenet et al. was a therapeutic trial for acute ABMR, we performed a sensitivity analysis of the risk ratio for graft and patient survival at 6 months by excluding it [19]. The sensitivity analysis revealed similar results with RRs of 1.01 (95% CI 0.97–1.06, I^2^ = 33%) and 1.01 (95% CI 0.98–1.04, I^2^ = 0%) for graft survival and patient survival, respectively [Figure S1 and Figure S2].

#### 3.2.2. Long-Term Graft and Patient Survival

Due to significant variance in the extended follow-up durations among the included trials, long-term outcomes were assessed via narrative synthesis, rather than quantitative pooling. Overall, the extended follow-up did not demonstrate a long-term graft or patient survival benefit associated with rituximab. After a median follow-up of 4.0 years, van den Hoogen et al., reported an uncensored graft survival of 79.7% in the rituximab group compared to 78.2% in the placebo group [20]. Patient survival at the end of follow-up was similarly comparable (87.0% vs. 85.9%, respectively) [20]. At 7 years post-treatment for active ABMR (mean follow-up 64.2 months), Bailly et al. reported no significant difference in death-censored graft survival (44% in the rituximab group vs. 55% in the placebo group, *p* = 0.91) [6]. Two deaths occurred in the rituximab group (one melanoma, one tuberculosis) compared to none in the placebo group [6].

In Tydén et al. (2012)’s three-year follow-up, 44 patients were available for follow-up in the rituximab group and 47 in the placebo group [24]. Graft loss occurred in one patient in the rituximab group due to chronic rejection, and one patient in the placebo group due to recurrence of the disease [24]. However, eight deaths were reported in the rituximab group compared to zero in the placebo group (8 vs. 0; *p* = 0.006), and six of the eight deaths were due to cardiovascular events (myocardial infarction or cardiac arrest). Notably, in the original paper by Tydén et al. (2009), one death was reported in each group, whereas the three-year follow-up (2012) reported no deaths in the placebo group, contradicting the previous publication [12,24]. Another discrepancy was observed in the original study, where the reported total in the rituximab group (*n* = 68) did not equal the sum of males and females in the same group (*n* = 46 + 23 = 69).

#### 3.2.3. Safety Profile and Risk of Diseases

At 6 months, bacterial and CMV infection in the rituximab group had overall RRs of 0.86 (95% CI 0.71–1.03, I^2^ = 0%) and 1.36 (95% CI 0.75–2.4, I^2^ = 0%), respectively (Figure S3 and Figure S4). Notably, leukopenia risk was statistically significant (*p* = 0.003) in the rituximab group, with an overall RR of 8.15 (95% CI 2.00–33.15, I^2^ = 0%) (Figure S5). In Bailly et al., seven cases of malignancy were observed in the rituximab group, with none in the placebo group [6]. In contrast, van den Hoogen et al. showed similar rates of malignancy between both groups [20]. Table 3 and Table 4 summarize the main reported outcomes of interest.

### 3.3. Analyzing Biases and Certainty of Evidence

**Figure 5:**
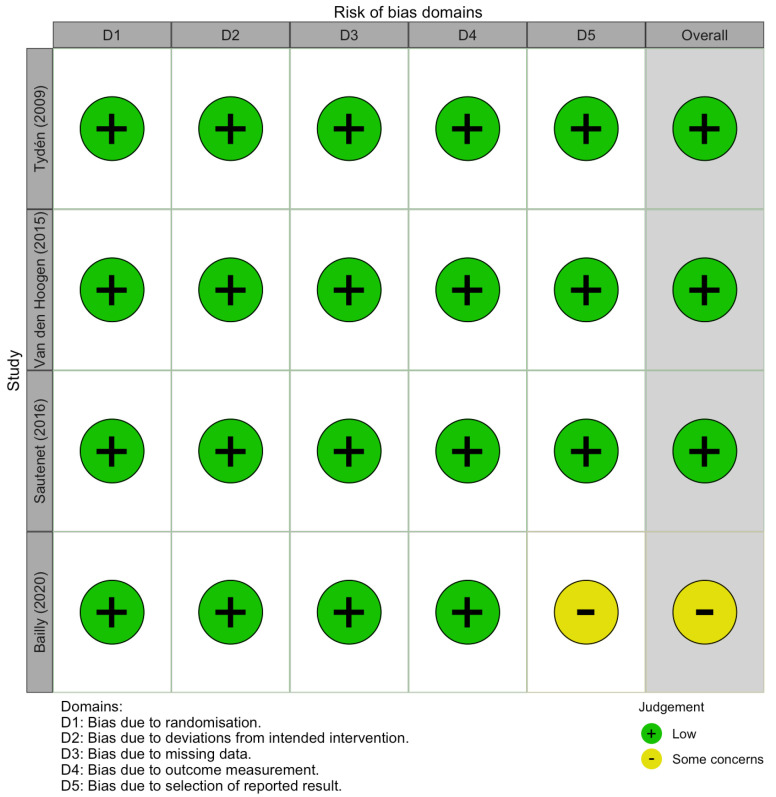
Risk of bias summary figure, using the revised Cochrane risk of bias methodology [6,12,19,20].

Given the limited number of trials, the evaluation of the publication bias did not provide a reliable assessment. Funnel plots assessing the publication bias of RCTs related to graft survival, patient survival, and infection rates in rituximab-treated recipients are presented in the Supplementary File. The risk of bias assessment for the included trials is shown in Figure 5. All RCTs were reported by using a proper randomization technique and were rated as having low risk, except Bailly et al., which was reported as having some concerns regarding its reporting. The certainty of evidence for each of the five reported outcomes, along with the relative effect of intervention, is presented in Table 5.

## 4. Discussion

In this systematic review and meta-analysis, we conducted a comprehensive search across five databases, strictly including studies that met our inclusion and exclusion criteria. The primary aim of this study was to evaluate whether rituximab would improve patient survival, graft survival, and acute rejection rates in ABO-compatible, non-sensitized renal transplant patients. We found low-certainty evidence suggesting that, compared to the placebo, rituximab did not show any additional benefit in either primary or secondary outcomes over both the short- and long-term follow-ups. Altogether, these findings suggest that rituximab alone may be insufficient as a standalone induction agent. Our results are consistent with previous findings reported in the meta-analysis by Cheungpasitporn et al. and the narrative review by Macklin et al. [28,29].

We observed a notable clinical heterogeneity among the included trials, which limits the interpretability of the pooled results. The included studies differed in their immunological risk, induction protocols, and primary and secondary outcome endpoints. Regarding immunological risk, Tydén et al. included low-risk patients (PRA ≤ 50%) and van den Hoogen et al. included moderate-risk patients (PRA < 85%), whereas the RITUX-ERAH trial included patients with active antibody-mediated rejection without a defined PRA threshold [6,12,19,20]. The induction protocol also varied between studies; the RITUX-ERAH trial utilized rituximab alongside IVIg and plasmapheresis as a therapeutic intervention, contrasting with the prophylactic use alongside standard induction agents in the other trials [6,12,19,20]. Follow-up durations ranged from 6 months to 7 years. The recent literature highlights the critical nature of these differences; Yilmaz et al. demonstrated that the efficacy of rituximab is dependent on the immunological risk profile and the timing of administration [30].

In terms of the safety profile, we found a potential concern regarding excessive immunosuppression. We observed a markedly increased risk of leukopenia among the rituximab group in prophylaxis settings. This profound leukopenia is of particular concern because it may predispose patients to opportunistic infections and may necessitate clinicians to reduce other important maintenance immunosuppressants, which may limit the efficacy of the overall treatment protocol. While no statistically significant difference was observed in the rates of bacterial and CMV infection between the two groups, a trend toward increased CMV infection was observed in the rituximab group, raising concerns about the safety and risk–benefit ratio. This aligns with Yamauchi et al., who found that early post-transplant rituximab use did not improve graft or patient survival but observed a trend toward higher rates of CMV and BK polyomavirus infection [31]. Furthermore, a trend toward higher malignancy rates in the rituximab group was observed in the RITUX-ERAH trial 7-year follow-up by Bailly et al. [6]. This may be due to the complete depletion of circulating B cells, resulting in profound immunosuppression and a long-term impairment of natural tumor immunosurveillance. However, establishing a causal link between rituximab and malignancy risk remains premature, as the evidence from the literature is contradictory. Two large studies by Emery et al. and Lopez-Olivo et al. reported no evidence of an increased risk of malignancy, but a large cohort by Tao et al. found an increase in the incidence of malignancy [32,33,34].

The standard dosing regimen may be directly related to the observed infection risk. All three included trials utilized the standard rituximab dose of 375 mg/m^2^ [6,12,19,20]. Some centers have implemented doses as low as 100 mg/patient in transplantation settings [35]. Okada et al. in particular demonstrated that a single dose of 100 mg rituximab was effective for preventing ABMR without increasing the risk of infection compared to standard protocols [36]. Additionally, a network meta-analysis found that induction with 200 mg of rituximab in ABO-incompatible patients showed no significant difference compared to 500 mg in terms of graft survival and biopsy-proven rejection; rather, the 200 mg dosage was associated with lower bacterial and BK virus infections, but CMV infection was similar between groups [7]. Although the use of prophylactic agents with rituximab have been shown to lower the incidence of some infections, their overall benefit–risk profile remains unclear and warrants further investigation [37,38]. Future trials are needed before revising the current dosing practice, but dose reduction appears to be a promising strategy to limit the common adverse effects.

Several potential mechanisms may explain these findings: First, rituximab specifically targets CD20+ cells, sparing plasma cells—important mediators of ABMR [19]. CD20 is expressed on B cells at all stages until they differentiate into plasma cells, where CD20 expression is downregulated [39]. Because mature plasma cells lack CD20 expression, they remain resistant to rituximab-mediated depletion. Consequently, these cells can continuously secrete alloantibodies, driving the rejection process despite the clearance of precursor B cells. Although van den Hoogen et al. found a statistically significant decrease in the levels of intragraft plasma cells following rituximab, there was no clinical improvement, suggesting that key effector cells that are critical in driving ABMR reside outside the graft [40]. Second, one major challenge with rituximab induction is the persistence of memory B cells (CD27+), which may contribute to long-term graft failure [41,42]. A study by Kamburova et al. found that a single dose of 375 mg/m^2^ rituximab induction failed to eliminate the CD27+ memory B cells, which are characterized by the high expression of pro-survival molecules [43]. Emerging therapies could explore the use of CD27 inhibitors as an adjunct to rituximab. However, this strategy must be carefully evaluated due to the potential adverse effects and the lack of clinical data on CD27+ inhibitors in renal transplantation. Third, the timing of the rituximab induction is likely to be of importance [44]. This is supported by a study conducted by Tydén et al. in which they observed higher rates of acute rejection in ABO-compatible patients (both adults and pediatrics) who received rituximab one day before transplantation, compared to the ABO-incompatible group who received rituximab 1 month prior to transplantation [45]. This suggests that earlier induction of rituximab may allow sufficient time for immunomodulation and reduce the risk of “cytokine storm”: a transient increase in cytokine release following rituximab induction, which was suspected in a prematurely halted trial by Clatworthy et al. due to an unexpected high incidence of graft rejection [44]. Notably, Clatworthy’s trial used a corticosteroid-free maintenance regimen, unlike other trials included in this review, possibly explaining these unexpected findings [6,12,44,46,47]. Fourth, while B cells are primarily known for enhancing immune response, some B cells have an immunoregulatory and atheroprotective function; the depletion of such B cells could cause the exacerbation of autoimmunity and atherosclerosis [48]. In terms of rituximab and its association with increased cardiovascular risk, the literature remains contradictory. A large propensity-matched study found that rates of atherosclerotic cardiovascular disease over an 8-year follow-up period were significantly lower in the rituximab group compared with the controls [49]. Tydén’s 3-year follow-up shows a significant increase in mortality among rituximab patients (8 vs. 0 deaths; *p* = 0.006); six of the eight patients’ deaths were due to cardiovascular events [24]. However, Tydén’s 3-year results should be interpreted with caution, due to reporting inconsistencies (see Section 3.2.2) [12,24]. Nevertheless, further studies exploring the relationship of rituximab with increased cardiovascular risk and specific immunomodulation approaches to preserve immunoregulatory B cells are warranted.

### 4.1. Recommendations

Standardized thresholds for inclusion criteria, such as DSA and PRA levels, and outcome reporting, such as rejection criteria and threshold for leukopenia, are crucial for improving inter-trial comparability and enabling a more robust meta-analysis. Rituximab’s lack of efficacy is often attributed to its poor plasma cell depletion in trials [6]. Thus, future trials with rituximab and plasma cell-directed therapy may be needed, as a randomized trial of 44 ABMR patients treated with bortezomib as a stand-alone induction agent showed no added benefit in terms of graft failure [50]. Complement proteins play a key role in the inflammatory process and transplant rejection [51]. C4d deposition in peritubular capillaries reflects classical complement pathway activation and is associated with an increased risk of graft loss [52]. Targeting the terminal complement pathway, such as with anti-C5 therapies, represents a promising approach for patients with ABMR [53]. Trials investigating the use of early complement inhibitors are still in their early stages, especially in the context of ABO-compatible and non-sensitized renal transplantation [51]. The preliminary data show that the C1 esterase inhibitor (C1-INH) may reduce inflammatory injury in allografts and improve histologic outcomes, even outside of overt rejection episodes [51]. Notably, the mechanism of C1-INH complements B-cell depleting agents like rituximab, which do not target downstream complement pathways. Together, this combination may provide synergistic protection in select cases where complement activation is suspected, but traditional risk markers are absent. However, no randomized controlled trials to date have evaluated C1-INH specifically in low-immunologic risk or ABO-compatible kidney transplant recipients, and its use in this population remains investigational.

### 4.2. Strengths and Limitations

To the best of our knowledge, this is the only systematic review that includes a long-term analysis (up to 7 years) of the efficacy and safety of rituximab. This study’s adherence to PRISMA guidelines further strengthens it [21]. Nevertheless, several limitations should be acknowledged. First, the low sample size limited the statistical power of the study, which could lead to missing important results. The inclusion of studies that used intention-to-treat analysis, such as the van den Hoogen et al. trial and the 1-year RITUX-ERAH trial by Sautenet et al., alongside those using per-protocol analysis, constituted a major source of heterogeneity [6,12,19,20,24]. Consequently, the certainty of evidence was low, and the interpretation of publication bias using funnel plots was limited due to the unreliability of visual inspection with fewer than ten studies [26]. However, this was inevitable because we strictly included fully published RCTs to ensure the highest level of evidence, whereas previous reviews included either unpublished or prematurely terminated studies [28,29]. Nevertheless, the limited sample size hinders the generalizability of our findings; thus, the results should be interpreted with caution.

## 5. Conclusions

Our meta-analysis found that the addition of rituximab to an immunosuppressive regimen did not produce a notable improvement in our primary and secondary outcomes. Although a slight reduction in biopsy-proven acute rejection (BPAR) was observed in the rituximab group, other factors, such as graft and patient survival, showed minimal to no change compared to the placebo group. Additionally, the potential risks associated with rituximab, such as increased CMV infections and leukopenia caused by excessive B cell depletion, should be considered. Further trials focusing on the use of rituximab in ABO-compatible, non-sensitized patients, especially in a large sample size as per the recommendations made in this study, are needed to draw a firm conclusion about the efficacy and safety of rituximab.

## Figures and Tables

**Table 1:** Main characteristics of included studies.

Variable	Tydén (2009) [12], Sweden	Van den Hoogen (2015) [20], The Netherlands	Sautenet (2016) [19] ^1^, France	Bailly (2020) [6] ^1^, France
Included patients: (Rit/ Control); inclusion PRA	136 (68/68); PRA ≤ 50%	280 (138/142); PRA < 85%	38 (19/19); PRA NA	38 (27/11); PRA NA
Study period (months) ^2^	6	24	12	84
Measured outcomes	Acute rejection, graft loss, or death	BPAR	Graft loss or absence of renal function improvement (at day 12)	Patient survival, graft loss, renal function
Primary analysis strategy ^3^	PP	mITT	ITT	PP
Rituximab dose	375 mg/m^2^, in 500 mL 5% Glucose	375 mg/m^2^, in 500 mL 0.9% Sodium Chloride	375 mg/m^2^	375 mg/m^2^
Placebo dose	500 mL 5% Glucose only	500 mL 0.9% Sodium Chloride only	Placebo (Not speci-fied)	(Not specified)
Post-transplant Immunosup-pressive regimen	Tacrolimus, MMF ^4^, Prednisolone	Tacrolimus, MMF, Prednisolone	ATG ^4^, Tacrolimus, MMF, or EC-MPS	ATG, Tacrolimus, MMF or EC-MPS
Prophylaxis regimen	Co-Trimoxazole, valganciclovir	Co-Trimoxazole, valganciclovir	NA	NA

^1^ Sautenet (2016) [19] and Bailly (2020) [6] are reports from the same randomized controlled trial (RITUX-ERAH) at different follow-up intervals. ^2^ Long-term follow-up duration varied among the included trials. Tyden et al. reported outcomes at a 3-year follow-up; van den Hoogen et al. reported a median follow-up of 4.0 years; and Bailly et al. (the RITUX-ERAH extension) reported a mean follow-up of 5.3 years (64.2 months). ^3^ ITT, intention-to-treat; PP, per-protocol; and mITT, modified intention-to-treat. ^4^ MMF = Mycophenolate Mofetil. ATG = Anti-Thymocyte Globulin. EC-MPS = Enteric-Coated Mycophenolate Sodium.

**Table 2:** Comparison of patient characteristics between the rituximab and placebo groups.

Variable	Tydén (2009) [12], Sweden		Van den Hoogen (2015) [20], The Netherlands		Sautenet (2016) [19] ^1^, France		Bailly (2020) [6] ^1^, France	
	Rituximab	Placebo	Rituximab	Placebo	Rituximab	Placebo	Rituximab	Placebo
Sample size	(*n* = 68)	(*n* = 68)	(*n* = 138)	(*n* = 142)	(*n* = 19)	(*n =* 19)	(*n* = 27)	(*n* = 11)
Age (yr)	51.3 ± 12.0	47.0 ± 13.4	50.8 ± 13.2	49.8 ± 12.3	44.6 ± 16.8	46.7 ± 16.2	48 ± 16	40 ± 15
Male sex (%)	51.11	48.88	69.6	63.4	42.1	68.4	55.6	54.5
First transplant (%)	100	91.17	NA	NA	52.6	68.4	63	54.5
Living donor (%)	27.94	36.76	58.7	57.0	0	5.3	0	9.1
Inclusion PRA	≤50%		<85%		NA		NA	

^1^ Sautenet (2016) [19] and Bailly (2020) [6] are reports from the same randomized controlled trial (RITUX-ERAH) at different follow-up intervals.

**Table 3:** Comparative outcomes of patients between the rituximab-treated and placebo groups.

Variable	Tydén (2009) [12], Sweden		Van den Hoogen (2015) [20] ^1^, The Netherlands		Sautenet (2016) [19] ^2^, France		Bailly (2020) [6] ^2^, France	
	Rituximab	Placebo	Rituximab	Placebo	Rituximab	Placebo	Rituximab	Placebo
Sample Size	(*n* = 68)	(*n* = 68)	(*n* = 138)	(*n* = 142)	(*n* = 19)	(*n* = 19)	(*n* = 27)	(*n* = 11)
Patient Survival ^1^ (%)	98.5	98.5	97.8\87.0	95.8\85.9	100	100	92.6	100
Graft Survival (%)	98.5	98.5	96.4\92.0	93.0\87.3	94.7	94.7	44	55
Bacterial Infection (%)	61.76	76.47	33.33\50.0	34.5\49.3	NA			
Fungal Infection (%)	4.41	7.35	16.7\22.4	19.7\25.4	NA			
CMV Infection (%)	4.41	1.47	14.5\15.9	11.3\12.7	NA			
BKV Infection (%)	1.47	5.88	NA	NA	NA			
Malignancies (%)	NA		5.8 (within 24 months)	5.6 (within 24 months)	NA			

^1^ Van den Hoogen et al.’s values are reported in the following format: 6-month\end-of-follow-up (median of 4.0 years). ^2^ Sautenet (2016) [19] and Bailly (2020) [6] are reports from the same randomized controlled trial (RITUX-ERAH) at different follow-up intervals.

**Table 4:** Per-protocol comparison of the number of adverse events in RITUXI-ERAH studies between the rituximab and control groups.

Variable	Sautenet (2016) [19] ^1^, France		Bailly (2020) [6] ^1^, France	
	Rituximab	Placebo	Rituximab	Placebo
Sample size	(*n* = 27)	(*n* = 11)	(*n* = 27)	(*n* = 11)
Pyelonephritis and UTIs ^2^	3	7	19	16
CMV ^2^	3	0	3	1
BKV ^2^	2	0	3	0
Malignancies ^2^ (*n*)	1	0	7	0

^1^ Sautenet (2016) [19] and Bailly (2020) [6] are reports from the same randomized controlled trial (RITUX-ERAH) at different follow-up intervals. ^2^ RITUXI-ERAH studies report the number of events instead of the number of incidences.

**Table 5:** GRADE summary of findings.

Outcome	BPAR at 6 Months (Prophylaxis Setting)	Graft Survival at 6 Months	Patient Survival at 6 Months
Illustrative Control Group Risk	200 per 1000	948 per 1000	969 per 1000
Relative Effect (95% CI)	RR 0.76 (0.50 to 1.15)	RR 1.01 (0.98 to 1.05)	RR 1.01 (0.98 to 1.04)
Certainty of The Evidence (GRADE)	LOW Downgraded due to: Very serious imprecision (−2) ^1^	LOW Downgraded due to: Very serious indirectness (−2) ^2^	LOW Downgraded due to: Very serious indirectness (−2) ^2^
No. of Participants (Studies)	416 (2 RCTs)	454 (3 RCTs)	454 (3 RCTs)

Explanations: CI: Confidence interval and RR: risk ratio. The certainty for all outcomes started at HIGH, as all included studies were RCTs. Publication bias could not be assessed due to the inclusion of fewer than 10 studies. ^1^ Very serious imprecision: Downgraded one or two levels due to a small number of total events and/or a wide 95% confidence interval that crosses the line of no effect. ^2^ Very serious indirectness: Downgraded one or two levels because the analysis combined studies of rituximab for two fundamentally different clinical indications (prophylaxis against rejection and treatment of active rejection), making the pooled estimate difficult to interpret for either group.

## Data Availability

All data generated or analyzed during this study are included in this published article [and its Supplementary Information Files].

## Supplementary Materials

The following supporting information can be downloaded at: https://www.mdpi.com/article/10.3390/medicina62040636/s1, Supplementary File S1: Forest plots, funnel plots, and search strategy. Supplementary File S2: PRISMA 2020 checklist [54].
