# Supplementary material for: The Role of Rituximab in ABO-Compatible Renal Transplantation: A Comprehensive Systematic Review and Meta-Analysis of Randomized Controlled Trials
Source: Medicina (Kaunas). 2026 Mar 27;62(4):636. doi: 10.3390/medicina62040636 (PMC13118092; doi:10.3390/medicina62040636)
Supplement: Supplementary file 1 [file medicina-62-00636-s001.zip › Supplementary S1.pdf]

Supplementary file S1

Title: The Role of Rituximab in ABO-compatible Renal Transplantation: A Comprehensive Systematic Review and Meta-Analysis of Randomized Controlled Trials

FOREST PLOTS:

S1. Sensitivity analysis forest plot of graft survival (at 6 months)

Study or Subgroup	Rituximab Events	Rituximab Total	Placebo Events	Placebo Total	Weight	Risk Ratio M-H, Random, 95% CI	Year
Tydén (2009)	67	68	67	68	59.9%	1.00 [0.96, 1.04]	2009
Van den hoogen (2015)	133	138	132	142	40.1%	1.04 [0.98, 1.10]	2015
**Total (95% CI)**		**206**		**210**	**100.0%**	**1.01 [0.97, 1.06]**	
Total events	200		199				

Heterogeneity: Tau² = 0.00; Chi² = 1.49, df = 1 (P = 0.22); I² = 33%
Test for overall effect: Z = 0.69 (P = 0.49)

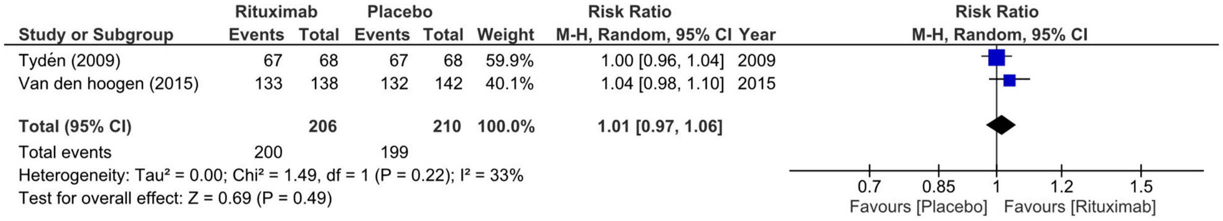

S2. Sensitivity analysis forest plot of patient survival (at 6 months)

Study or Subgroup	Rituximab Events	Rituximab Total	Placebo Events	Placebo Total	Weight	Risk Ratio M-H, Random, 95% CI	Year
Tydén (2009)	67	68	67	68	51.8%	1.00 [0.96, 1.04]	2009
Van den hoogen (2015)	135	138	136	142	48.2%	1.02 [0.98, 1.07]	2015
**Total (95% CI)**		**206**		**210**	**100.0%**	**1.01 [0.98, 1.04]**	
Total events	202		203				

Heterogeneity: Tau² = 0.00; Chi² = 0.56, df = 1 (P = 0.45); I² = 0%
Test for overall effect: Z = 0.68 (P = 0.50)

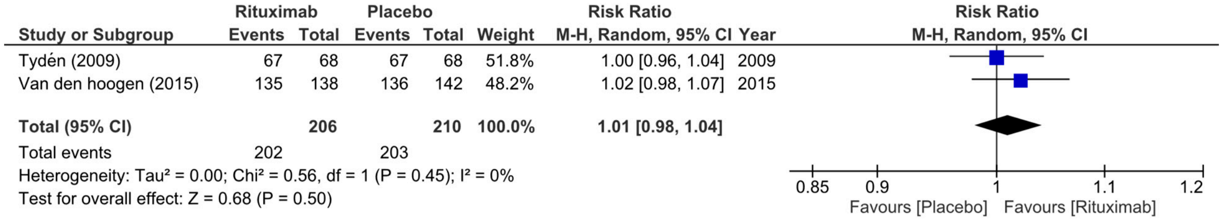

S3. Forest plot of bacterial infection (at 6 months)

Study or Subgroup	Rituximab Events	Rituximab Total	Placebo Events	Placebo Total	Weight	Risk Ratio M-H, Random, 95% CI	Year
Tydén (2009)	42	68	52	68	67.2%	0.81 [0.64, 1.02]	2009
Van den hoogen (2015)	46	138	49	142	32.8%	0.97 [0.70, 1.34]	2015
**Total (95% CI)**		**206**		**210**	**100.0%**	**0.86 [0.71, 1.03]**	
Total events	88		101				

Heterogeneity: Tau² = 0.00; Chi² = 0.88, df = 1 (P = 0.35); I² = 0%
Test for overall effect: Z = 1.62 (P = 0.11)

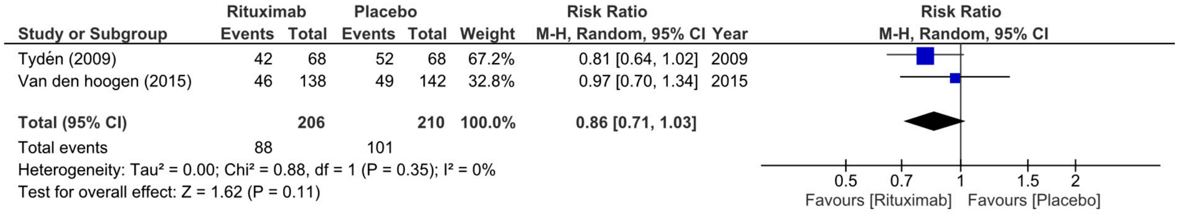

S4. Forest plot of CMV infection (at 6 months)

Study or Subgroup	Rituximab Events	Rituximab Total	Placebo Events	Placebo Total	Weight	Risk Ratio M-H, Random, 95% CI	Year
Tydén (2009)	3	68	1	68	7.0%	3.00 [0.32, 28.13]	2009
Van den hoogen (2015)	20	138	16	142	93.0%	1.29 [0.70, 2.38]	2015
**Total (95% CI)**		**206**		**210**	**100.0%**	**1.36 [0.75, 2.47]**	
Total events	23		17				

Heterogeneity: Tau² = 0.00; Chi² = 0.51, df = 1 (P = 0.47); I² = 0%
Test for overall effect: Z = 1.03 (P = 0.30)

Risk Ratio
M-H, Random, 95% CI

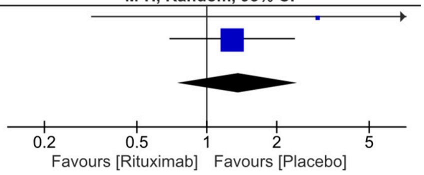

S5. Forest plot of leukopenia incidence (at 6 months)

Study or Subgroup	Rituximab Events	Rituximab Total	Placebo Events	Placebo Total	Weight	Risk Ratio M-H, Random, 95% CI	Year
Tydén (2009)	3	68	1	68	33.1%	3.00 [0.32, 28.13]	2009
Van den hoogen (2015)	26	138	2	142	66.9%	13.38 [3.24, 55.29]	2015
**Total (95% CI)**		**206**		**210**	**100.0%**	**8.15 [2.00, 33.15]**	
Total events	29		3				

Heterogeneity: Tau² = 0.24; Chi² = 1.26, df = 1 (P = 0.26); I² = 21%
Test for overall effect: Z = 2.93 (P = 0.003)

Risk Ratio
M-H, Random, 95% CI

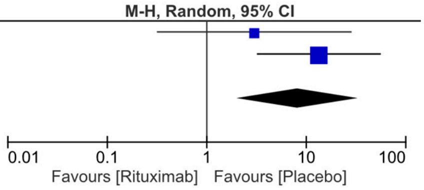

FUNNEL PLOTS (PUBLICATION BIAS):

S6. Funnel plot of biopsy-proven acute rejection (at 6 months)

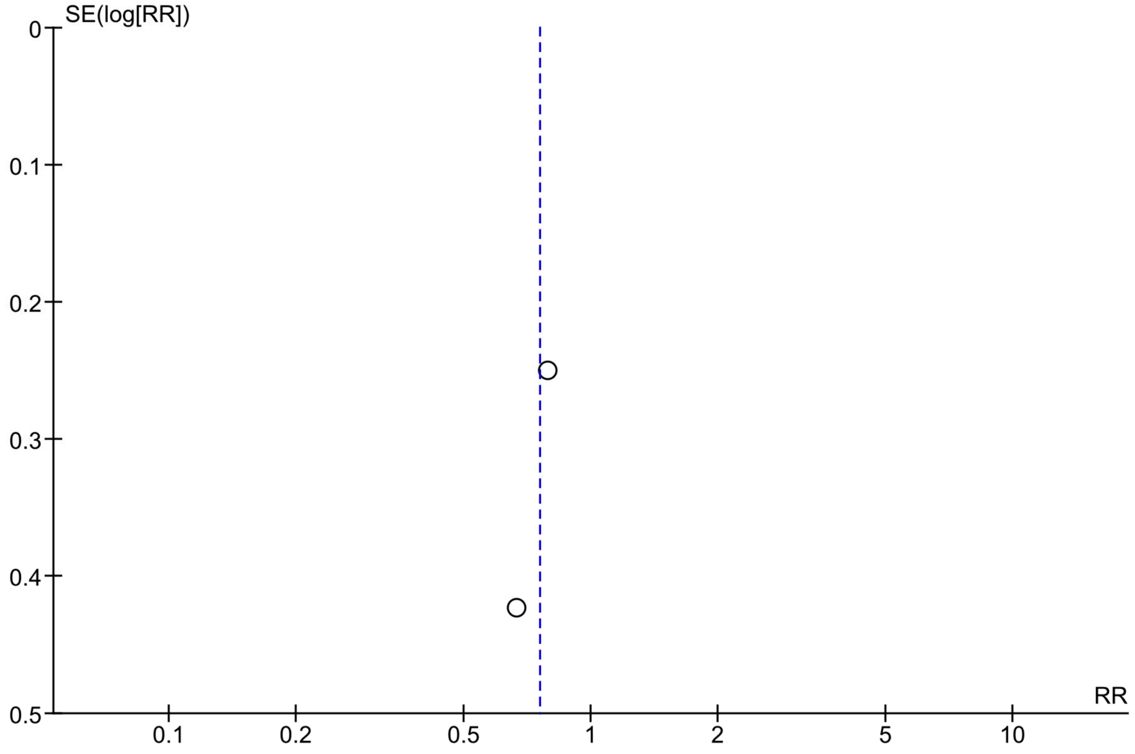

S7. Funnel plot of graft survival (at 6 months)

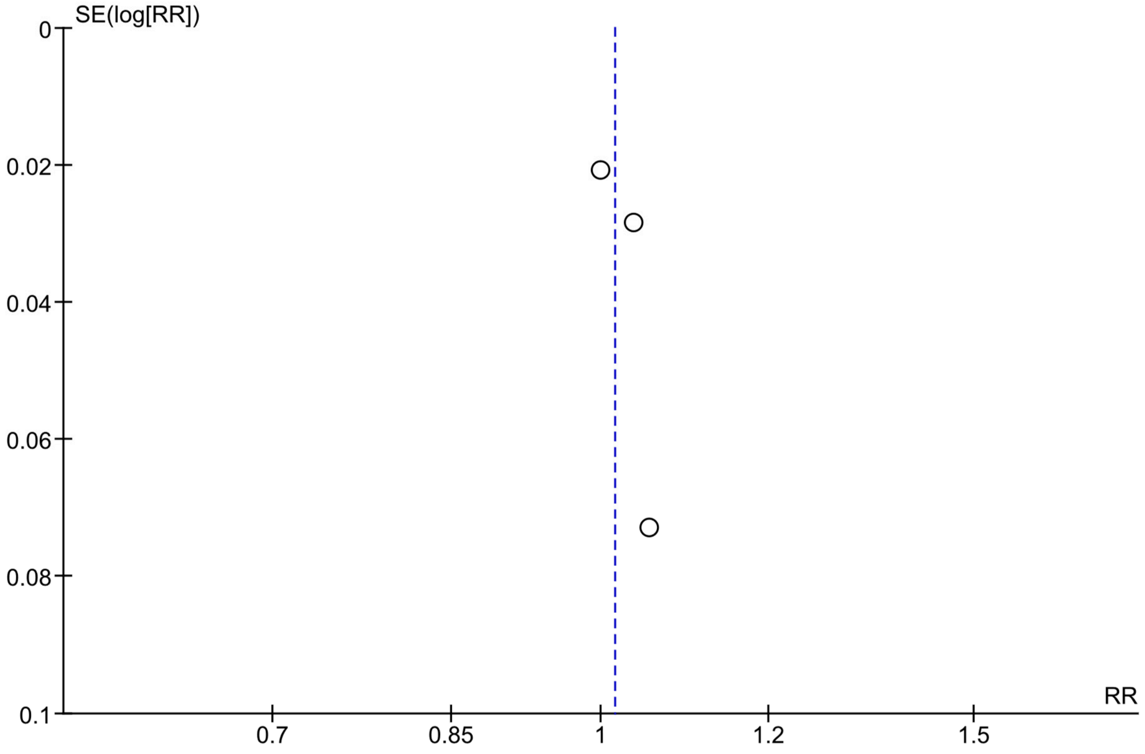

S8. Funnel plot of patient survival (at 6 months)

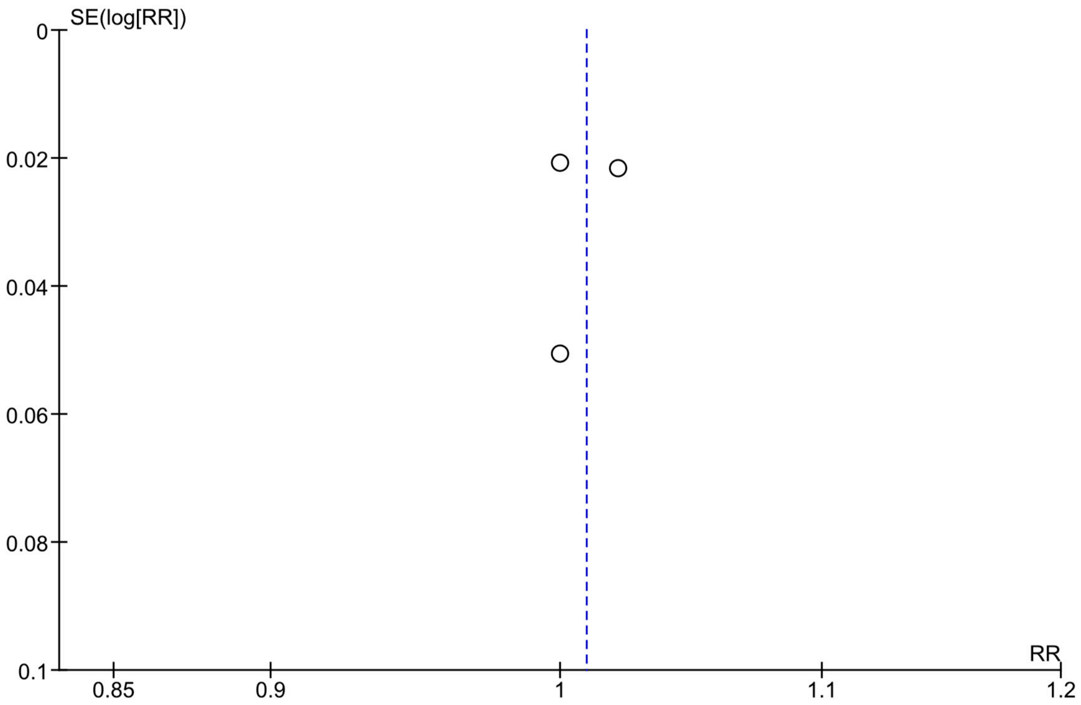

S9. Funnel plot of Bacterial infection (at 6 months)

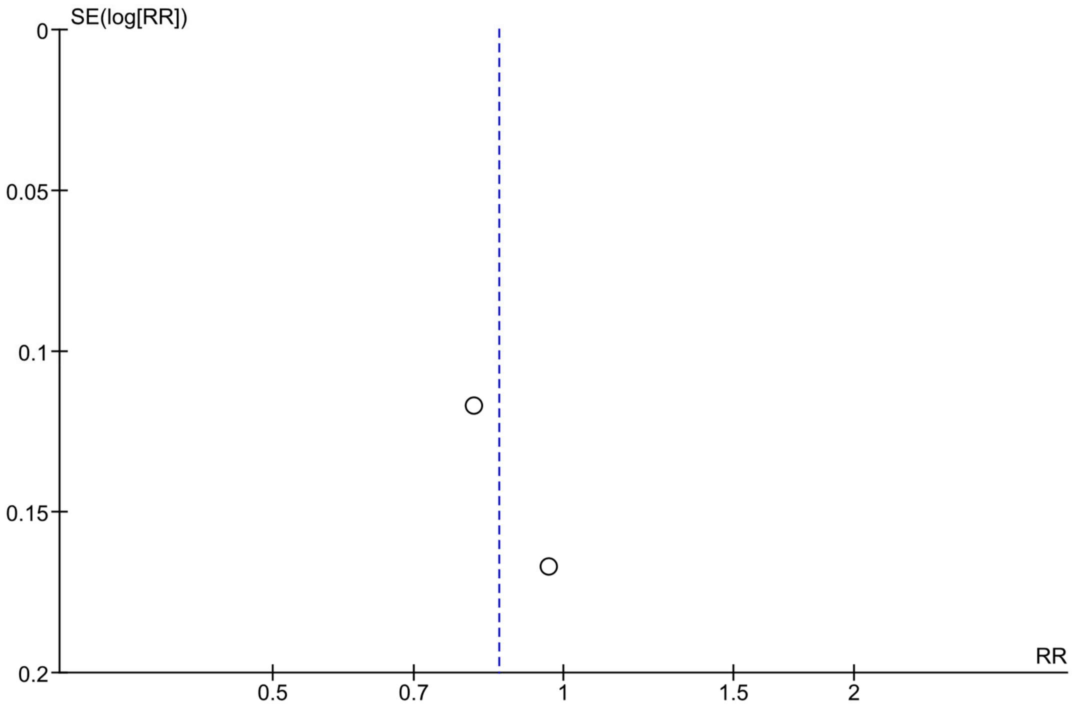

S10. Funnel plot of CMV infection (at 6 months)

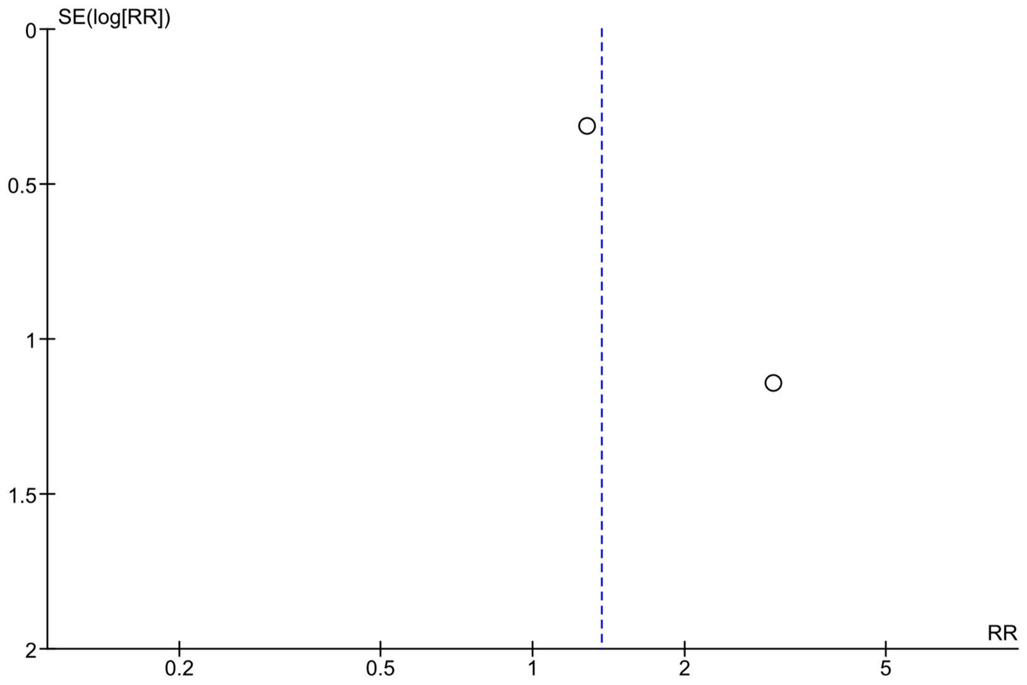

S11. Funnel plot of leukopenia incidence (at 6 months)

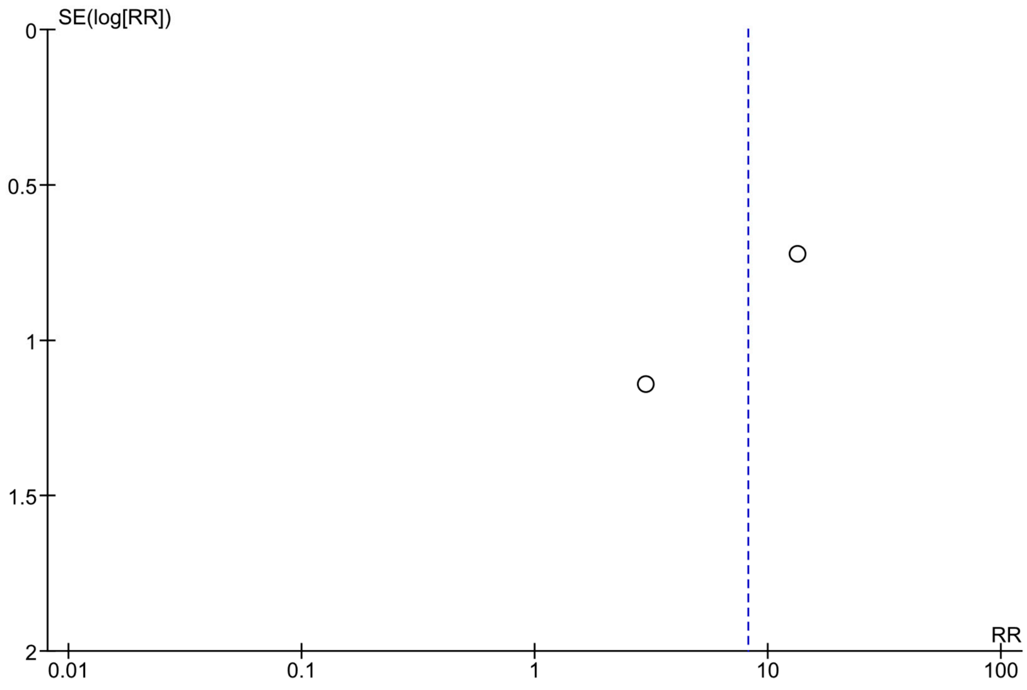

SEARCH STRATEGY:

Google Scholar: ("Rituximab" OR "anti-CD20" OR "B-lymphocyte depleting agent") AND ("Induction therapy" OR "Immunosuppression" OR "Pre-transplantation treatment") AND ("Renal transplantation" OR "Kidney transplant" OR "Graft survival")

PubMed: ("Rituximab" OR "anti-CD20" OR "B-lymphocyte depleting agent") AND ("Induction therapy" OR "Immunosuppression" OR "Pre-transplantation treatment") AND ("Renal transplantation" OR "Kidney transplant" OR "Graft survival")

Ovid MEDLINE: ("Rituximab" OR "anti-CD20" OR "B-lymphocyte depleting agent") AND ("Induction therapy" OR "Immunosuppression" OR "Pre-transplantation treatment") AND ("Renal transplantation" OR "Kidney transplant" OR "Graft survival")

Web of Science: ("Rituximab" OR "anti-CD20" OR "B-lymphocyte depleting agent") AND ("Induction therapy" OR "Immunosuppression" OR "Pre-transplantation treatment") AND ("Renal transplantation" OR "Kidney transplant" OR "Graft survival")

ScienceDirect: ("Rituximab" OR "anti-CD20" OR "B-lymphocyte depleting agent") AND ("Induction therapy" OR "Immunosuppression" OR "Pre-transplantation treatment") AND ("Renal transplantation" OR "Kidney transplant" OR "Graft survival")

ClinicalTrials.gov: ("Rituximab" OR "anti-CD20" OR "B-lymphocyte depleting agent") AND ("Induction therapy" OR "Immunosuppression" OR "Pre-transplantation treatment") AND ("Renal transplantation" OR "Kidney transplant" OR "Graft survival") + Manual Search

ISRCTN: ("Rituximab" OR "anti-CD20" OR "B-lymphocyte depleting agent") AND ("Induction therapy" OR "Immunosuppression" OR "Pre-transplantation treatment") AND ("Renal transplantation" OR "Kidney transplant" OR "Graft survival") + Manual Search

ICTRP: ("Rituximab" OR "anti-CD20" OR "B-lymphocyte depleting agent") AND ("Induction

therapy" OR "Immunosuppression" OR "Pre-transplantation treatment") AND ("Renal transplantation" OR "Kidney transplant" OR "Graft survival") + Manual Search
